# Breathable and Stretchable Temperature Sensors Inspired by Skin

**DOI:** 10.1038/srep11505

**Published:** 2015-06-22

**Authors:** Ying Chen, Bingwei Lu, Yihao Chen, Xue Feng

**Affiliations:** 1 Department of Engineering Mechanics, Tsinghua University, Beijing 100084, China; 2 Center for Mechanics and Materials, Tsinghua University, Beijing 100084, China

## Abstract

Flexible electronics attached to skin for healthcare, such as epidermal electronics, has to struggle with biocompatibility and adapt to specified environment of skin with respect to breath and perspiration. Here, we report a strategy for biocompatible flexible temperature sensors, inspired by skin, possessing the excellent permeability of air and high quality of water-proof by using semipermeable film with porous structures as substrate. We attach such temperature sensors to underarm and forearm to measure the axillary temperature and body surface temperature respectively. The volunteer wears such sensors for 24 hours with two times of shower and the ***in vitro*** test shows no sign of maceration or stimulation to the skin. Especially, precise temperature changes on skin surface caused by flowing air and water dropping are also measured to validate the accuracy and dynamical response. The results show that the biocompatible temperature sensor is soft and breathable on the human skin and has the excellent accuracy compared to mercury thermometer. This demonstrates the possibility and feasibility of fully using the sensors in long term body temperature sensing for medical use as well as sensing function of artificial skin for robots or prosthesis.

Mark Weiser, the father of ubiquitous computing, claimed that the computing technology in 21^st^ century will be disappeared in a way that we are freed to use them without thinking and noticing. With this concept wearable electronics aimed at health management and healthcare improvement, is booming and flourishing scientifically and commercially. Flexible sensors that can be conformal and mechanical-invisible are the kernel of the wearable electronics. Epidermal electronics introduced in 2011 is convinced to have the potential to be applied to a large variety of medical aspects^1^, such as skin temperature measuring^2^ , skin hydration and strain determination^3^,^4^,^5^, the sensing of electrophysiological (EP) signals including electrocardiograms (ECG) and electromyograms (EMG)^6^,^7^,^8^, as well as the nanogenerator powering wearable electronics^9^ and so on. By integrating electronic circuits, sensors and/or communication components to ultrathin, lightweight and stretchable membranes like thin silicone and polyimide (PI) film, the epidermal electronics is mechanically disappeared to the wearer^10^.

Body temperature is the basal index in physiology, as temperature affects the rate of the chemical reactions in physiological activities. Real-time measuring temperature greatly improves the healthcare quality of new-born babies or patients in anesthesia, whose thermoregulation mechanism is unsound. In traditional medical care, axillary temperature is measured by the mercury thermometer. However, such way is inconvenient for babies because babies cannot stably keep the mercury thermometer in underarm according to the requirement. Therefore the demanding for a wearable and comfortable temperature sensor with enough accuracy which can attach to the human skin for several days is strongly desired. Besides, with ultra-flexible and excellently biocompatible temperature sensor, continuous body temperature measuring can be realized to reflect emotion changes, which can be used in physiological research and chronobiology study; female menstrual cycle, which can be used to predict ovulation time, thus increase pregnancy rate; and more generally predict disease and monitor postoperative recovery and so on^11^. In practice, it helps to build the smart medical and healthcare system^12^. Many efforts have been made in flexible and wearable temperature sensor. Flexible brain core temperature sensor based on flexible circuits and materials fixed with an elastic textile headband is used to measure the brain temperature^13^. Discrete temperature sensor chips integrated to flexible copper-polyimide (PI) substrate as sensing cells can remain its functionality when bended to a 4 mm radius^14^. More advanced is the MEMS based technology with flexible polyimide as substrate to realize the flexibility of the temperature sensors^15^,^16^,^17^,^18^,^19^. Instead of relying on the substrate to gain flexibility, making the functional material flexible by doping them with polymer matrix is also investigated. High sensitive flexible temperature sensor based on Ni microparticle-filled polymer composite is designed to monitor body temperature with an RFID antenna system^20^. Similarly, graphite dispersed in polydimethylsiloxane (PDMS) with polyimide (PI) films as substrates is used to fabricate flexible temperature sensor arrays^21^. Besides resistive temperature sensor, other mechanism like pyroelectric^22^, cellulose-polypyrrole nanocomposite^23^ and organic semiconductor^24^ flexible temperature sensors are also developed. In the aspect of commercial, products of wireless thermometer (WT702, raiing) aiming at basal body temperature (BBT) measuring and recording have emerged, which count on the designed curvature of the morphology to reduce the incommodity during wearing. Although many efforts are made to fabricate many kinds of flexible temperature sensors, none of them are stretchable and biocompatible enough to conform to human body like skin that performs evaporation control and acts as water resistance. Therefore, such devices attached to skin result into skin allergy or infection due to air impermeability, or are prone to be destroyed by the liquid water due to lack of water-proof in wet environment such as body shower or sweat.

Here we present an ultra-flexible, stretchable and biocompatible temperature sensor (BCTS) fabricated by integrating temperature sensitive material with semipermeable polyurethane film (Opsite, Smith & Nephew) through transfer printing in solution, which is targeting at human body temperature measuring in a 24/7 way. Calibration in water bath gives the temperature resistance coefficient of this device, i.e., TRC = 0.002778 °C^–1^. Axillary’s temperature (i.e. the underarm temperature) measured in succession by *in vitro* testing with results referring to mercury thermometer indicates the practical application of this device in body temperature monitoring. And precise temperature changes on skin surface caused by flowing air of respiration and water dropping are also measured by locating the sensor to the forearm. The microstructure on the substrate is investigated by scanning electronic microscopy (SEM), which primarily endows the device with biocompatibility physically. 24 hours’ wearing demonstrates the biocompatible and safe property of the device subsequently. Moreover, changing the functional material and pattern of the device, this strategy including transfer-printing in solution is applicable for fabricating other flexible biocompatible electronic devices with different function.

## Results

**Figure 1:**
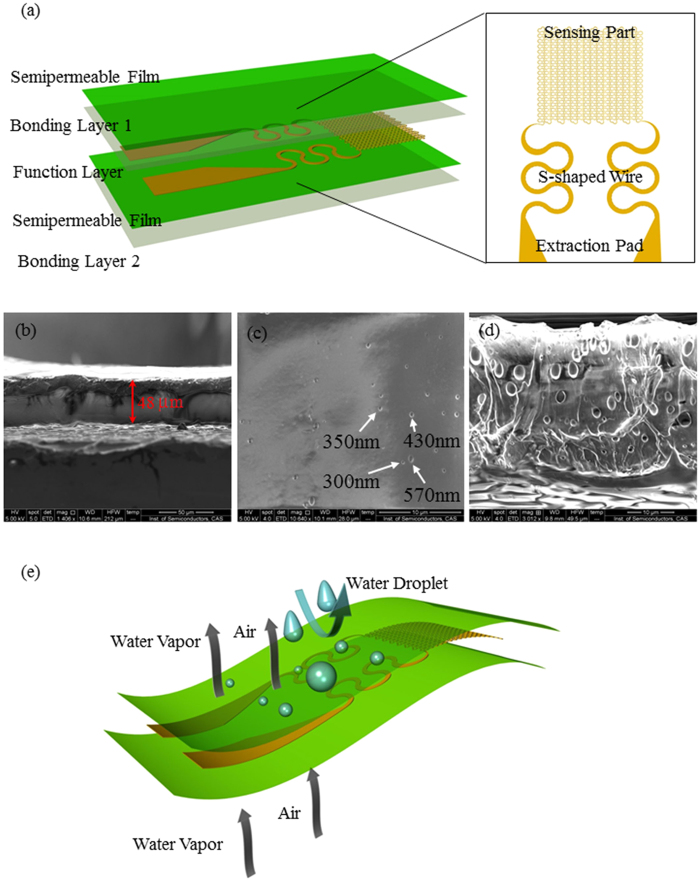
Illustration of the design and function of the biocompatible and stretchable temperature sensor. (**a**) Structure of the ultra-flexible temperature sensor on breathable film. (**b**) SEM image of the semipermeable film surface. (**c**) Cross section of the semipermeable film in SEM, (the film is pressed on the side face of the sample stage). (**d**) Micro-structure of the polyurethane layer (upper side) of the semipermeable film in SEM. (**e**) The illustration of water proof and vapor permeable property of the sensor.

The design of BCTS proposed here is based on the basic structure of semipermeable film (SF) encapsulation/ bonding layer/ function layer/ SF substrate/ adhesive layer as shown in Fig. 1(a). The function layer composed of the temperature sensing part, the S-shaped interconnects and extraction pads is located in the neutral plane of the whole device to minimize the strain caused by bending deformation. The SF substrate in the bottom not only acts as soft substrate but also separates sensing part from the skin, thus the signal won’t be affected by perspiration. The SF encapsulation on the top not only protects the sensor from environment influence but also puts the function layer in the neutral plane. The bonding layer is used to strengthen the integration between the function layer/substrate and the encapsulation layer, which determines the robustness of the device to a certain extent^25^. The adhesive layer is used to help the device intimately and conformal integrating with the skin surface for better signal and longer service life. Yet, if the thickness of the whole device is thin enough to adhere to the skin with only van der Waals force, the adhesive layer can be removed from the design. For the sake of shielding noise caused by piezoresistive effect and minimizing the stress and strain, the sensing part is designed into a serpentine line of gold arranged in palisade structure, in that stretching and compression deformation can be accommodated by the buckling or rigid body rotation of the serpentine lines^26^,^27^,^28^,^29^. Besides, compared with the fence style pattern^2^, the pattern used in the proposed sensor has an advantage that it is stretchable in two directions, rather than the traditional one direction. The width of the serpentine line which acts as sensing part is 10 *μm*, much smaller than the interconnects and pad ensuring that the resistance of this part is far larger than the other parts in the function layer (
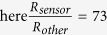
), which guarantees that the resistance change i.e., the temperature signal, of the function layer primarily generated by the sensing part.

The flexibility and stretchability, especially the biocompatibility heavily depends on the semipermeable film and the bonding of the sensing parts to the substrate. The SF used in this device (thickness ~ 40 *μm*) is a porous film (Opsite, Smith & Nephew) based on polyurethane with its cross section shown in the SEM image in Fig. 1(b). Figure 1(c,d) show the zoomed in SEM image of the surface and the cross section of the porous film, respectively, with pore sizes range from a few tens nanometer to several micrometer. The pore sizes are larger than the size of the air and water vapor molecule, yet smaller than that of the liquid water drop and bacteria. This enables the device to be permeable for the perspiration to go out as water vapor to avoid maceration and outside gas (such as O_2_) to get in and out for breathing, while being water proof overall to protect the function layer from short circuit. Therefore, the porous SF, impermeable to bacteria and water and permeable to water vapor and air, is physically biocompatible when applied to the human skin. For BCTS the nano pores in the film provide channels for redundant perspiration releasing to prevent epidermal tissue from maceration, while keeping the water in the external environment outside as shown in the illustration in Fig. 1(e). Quantitatively speaking, the water vapor transmission rate (WVTR, defined as the steady flow of water vapor per unit area of surface in unit time at a specified humidity and temperature^30^) of the SF is ~800 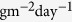 tested by ASTM (American Society for Testing Materials) E96-98^31^,^32^,^33^,^34^. And the WVTR of the normal skin is reported to be 200–500 gm^−2^day^−1^ 31,35,36, thus, despite the existence of the device, the skin under it can breathe normally. Consequently, the device can be left in place for 7 days when considering the biocompatibility alone. The bonding and adhesive layer are based on the hypoallergenic polyvinylethylether, which enables the device to be biocompatible to avoid the allergic reactions when attached to the human skin for a long time. Besides the strong bonding layer of the SF encapsulation keeps the gold sensing part from debonding, and that of the bottom film protect the device against delamination from the skin when in use. On the mechanical aspect, the similar or even smaller modulus of the semipermeable film (4.68 MPa^37^) to the modulus of normal human skin (4.6–20 MPa^38^) as well as the excellent elongation (600–700%) enables the device to be mechanically invisible to the user. FEM gives the equivalent tensile stiffness per unit width of the S shaped gold layer (0.7 N), which is negligible compared to that of the encapsulation and substrate (470 N). Therefore, the equivalent tensile stiffness of the system is mainly determined by the encapsulation and substrate layer, and so does the equivalent bending stiffness of the device. Thus the BCTS with feature of ultra-thin, flexible, stretchable, vapor permeable and water proof is extremely physically comfortable for 24/7 wearing. Moreover, changing the design of the function layer, the device structure introduced above and the fabrication process following are of value for a range of new devices based on biocompatible and breathable feature.

**Figure 2:**
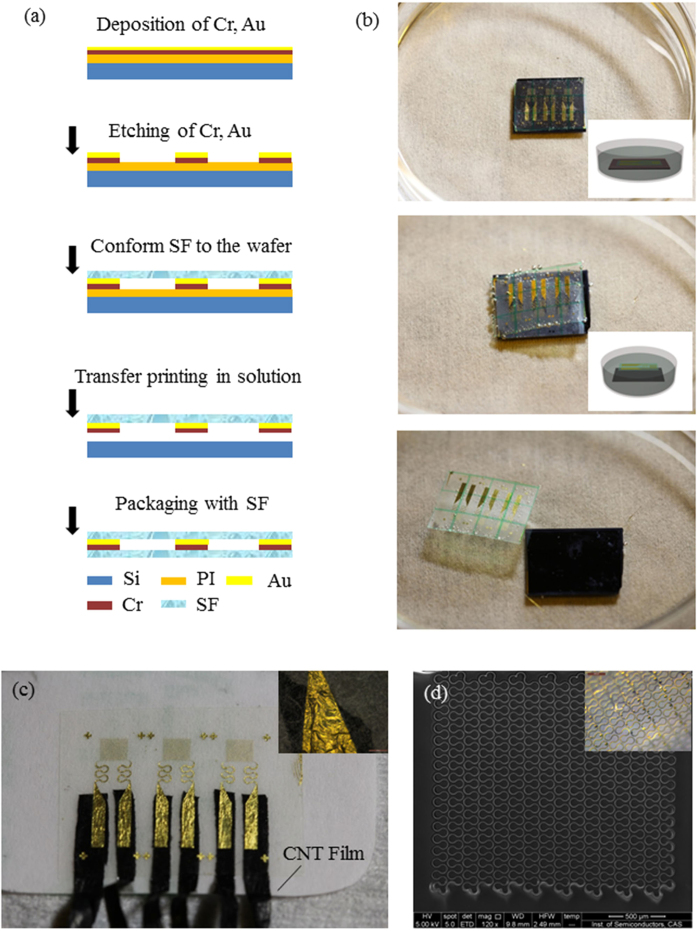
Fabrication of the biocompatible and strechable temperature sensor. (**a**) Fabrication flow chat.(**b**) Transfer-printing in solution. (**c**) The stretchable sensors on the glass sheet with packaging, the inset is the image of the CNT film contacting with the pad. (**d**) The SEM image of the sensor, the inset is the optical microscopy image of the sensor under bending deformation.

The fabrication process of BCTS, shown in Fig. 2(a), starts with preparation of sacrificial layer of polyimide (PI, ZKPI-3051), in which it is spinning coated on the silicon wafer with the thickness of ~1.5 *μm*, and cured at the temperature of 80 °C for 10 min, 120 °C for 10 min and 140 °C for 30 min to dispel the liquid in it. The curing under glass transition temperature enables it to bear the procedure following, yet remaining solubility in alkali solution. And the quality of film deposition is depended on the roughness of the sacrificial PI layer, which is influenced by the curing procedure^39^,^40^. On the PI covered silicon wafer Cr (10 nm)/Au (100 nm) are deposited sequentially, in which Cr acts as transition layer and Au the material in the function layer. Then etch Au/Cr into designed patterns by lithography, and conform the semipermeable film to the patterned wafer as soft substrate before transfer printing in solution. Transfer printing of a single layer of 100 nm thin metal film with complicated patterns is quite challenging for stamp-based transfer printing^41^,^42^,^43^, since fracture or wrinkles may occur in the thin metal film being pressed due to the Poisson effect of the stamp. Without pressing by the polymer stamp, transfer printing in solution offers a solution for pick-up and printing of such ultra-thin film. In fact, due to hydrostatic pressure and supporting of the soft targeting substrate, the ultra-thin film can keep flat and wrinkle-free. Here, KOH solution is used to undercut the sacrificial layer, i.e. PI, thus release the functional layer from the silicon wafer tenderly as a whole while printing which to the soft substrate, as shown in Fig. 2(b). An extra layer of relatively stiffer polymer film with green grids on it is used to hold the thin and soft substrate, keeping it from crimping in the etching solution/ DI water after releasing from the wafer or while washing the etching solution off it. Thus, the deformation of the device during transfer printing can be minimized to a large degree by this strategy.

Delamination of the inorganic thin film/organic soft substrate interface as well as the fracture of the thin film or the conjunction with the extraction wire shall lead to the failure of stretchable electronic devices. According to the slippage and delamination models of interfacial failure, the thinner the film the larger the critical interfacial failure strain^44^,^45^. Considering the small thickness of the film as well as the bonding layer between the thin film and the substrate introduced in this device, the failure of this device is prone to the fracture in the extraction pad at the conjunction of extraction wire when under stretching. Theoretical analysis indicates that the smaller the modulus and thickness of the extraction wire, the smaller the stress in the extraction pad, which is discussed in detail in Method Section. In the theoretical analysis, the pad and wire are regarded as a combination connected in series. According to the analysis, the stress in the pad is in linear proportion to the Young’s modulus and cross sectional area of the extraction wire. With comparable thickness, the Young’s modulus of the polymer based CNT composite thin film (~2 GPa, h = 10 μm, provided by Suzhou Institute of Nano-Tech and Nano-Bionics, China) is much smaller than that of the traditional silver glue (40 GPa)^46^. Thus, using polymer based CNT composite thin film ribbon as extraction wire can reduce the stress in extraction pad (to ~5%) by deforming to absorb certain energy when the device is under stretching to avoid fracture.

After encapsulated with SF, the final device is shown in Fig. 2(c), with the typical detail of the extraction wire based on CNT film ribbon connecting to the Au pad is shown in the inset, in which the existence of the extraction wire causes no obvious deformation in the Au pad. And tailing the bottom layer of semipermeable film into fillet at the corner helps to prevent delamination when attached to the human skin. The SEM image shown in Fig. 2(d) demonstrates the sensing part on the soft substrate, and the inset shows the sensor in bending configuration. As can be seen from the SEM image, this method of transfer printing in solution is quite effective for ultrathin devices to keep their integrity.

The function mechanism for the BCTS to measure temperature is based on temperature resistance effect of metal, i.e., the metal resistance changes when temperature changes. The temperature coefficient of resistance (TCR), denoted as *α*, describes the linear relationship the resistance *R* versus temperature *T*, i.e., 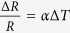. Therefore, when in application, the measured temperature by the BCTS can be calculated from the resistance change rate, i.e., 
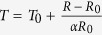
 where subscript 0 represents initial or reference value.

**Figure 3:**
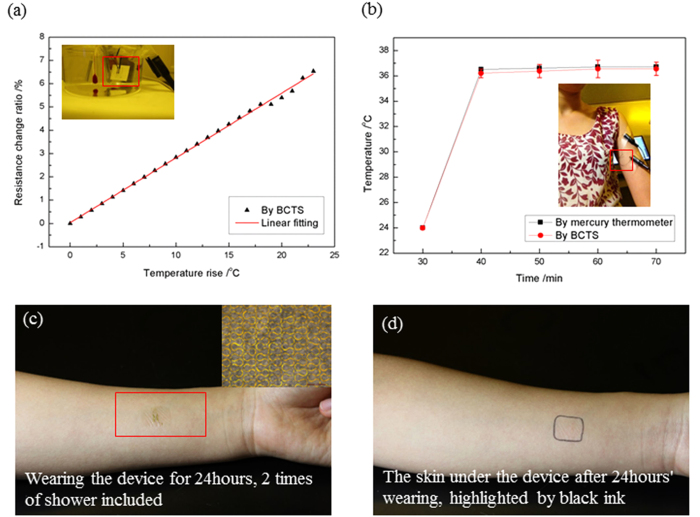
Performance of the biocompatible and stretchable temperature sensor. (**a**) Calibration results with inset showing the calibration setting. (**b**) In vitro test of the sensor and its comparison with the mercury thermometer. (**c**) Wearing the device for 24 hours with 2 times of shower. (**d**) The skin under the device after 24 hours’ wearing, no sign of maceration or stimulation is observed.

The calibration of the BCTS is carried out by water bath on a hot plate. The liquid environment is created by boiled DI water in order to exclude the influence or disturbing of the air bubble (generated while heating) to the temperature and resistance measuring. Resistance temperature detector (RTD, CENTER 376, Taiwan) based on platinum (Pt) is employed as temperature reference, shown in the inset of Fig. 3(a), and the device is fixed on a thin plate as highlighted by the red box. The DC resistance meter (TH2515, China) is used to measure and record the resistance of the device. The calibration temperature changes from 22 to 45 °C, which is far beyond the human body temperature variation range. The typical temperature rise and resistance change ratio relationship of this device is shown in Fig. 3(a), whose slope represents the TCR of this device. Linear fitting gives the TCR of the BCTS as ~0.002778 °C^−1^ showing good linearity of the device with respect to temperature. The response rate of the sensor is faster than the traditional kerosene thermometer, which is put next to the BCTS as another temperature reference as can be seen from the inset in Fig. 3(a). If compared with mercury thermometer the advantage will be even more obvious, thus the nursing time can be saved when apply this device to clinic.

With the TCR revealed in the calibration, continuous *in vitro* test of the BCTS is conducted on the volunteer with room temperature of ~24 °C. We select the position of underarm to conduct the *in vitro* test to demonstrate the performance of BCTS. Usually, underarm skin is non-planar and stretchable, and is prone to produce sweat due to sweat glands. During the *in vitro* test, the BCTS is in conformal attachment to the underarm skin, where body temperature can be measured with the least external convective influence, as shown in the inset in Fig. 3(b) with device highlighted in red box. The Resistance of the BCTS is measured and recorded every 10 min in 40 min, and transferred into temperature signal with TCR, as demonstrated in Fig. 3(b). Meanwhile, the mercury thermometer is used to take the body temperature of the volunteer in the same frequency at the same location as a comparison and reference. The test results of the clinical used mercury thermometer and that of the BCTS show good agreement, indicating that applying BCTS to measure the axillary temperature continuously is feasible and reliable.

Evaluation of biocompatibility of the device in long-term wearing is demonstrated by wearing the device in the forearm for the convenience in observation and display. The device is kept on the volunteer’s forearm for 24 hours, during which 2 times of shower is conducted. Figure 3(c) shows the device after 24 hours’ wearing, and the inset displays the microscopy image of the sensing part of the device immediately after being peeled off the skin. Attributing to the narrow width and serpentine shape design of the sensing part, as well as the neutral plane location, no obvious fracture or rupture is observed. The skin under the device after 24 hours’ wearing, whose outline is sketched in black ink in Fig. 3(d), shows neither sign of maceration or stimulation nor any significant difference to the normal area around it. Therefore, owing to features of porosity structure, small thickness and modulus, together with excellent elongation the device possesses good biocompatibility to the human skin, which guarantees the design of this device to be 24/7 wearable in health monitoring.

**Figure 4:**
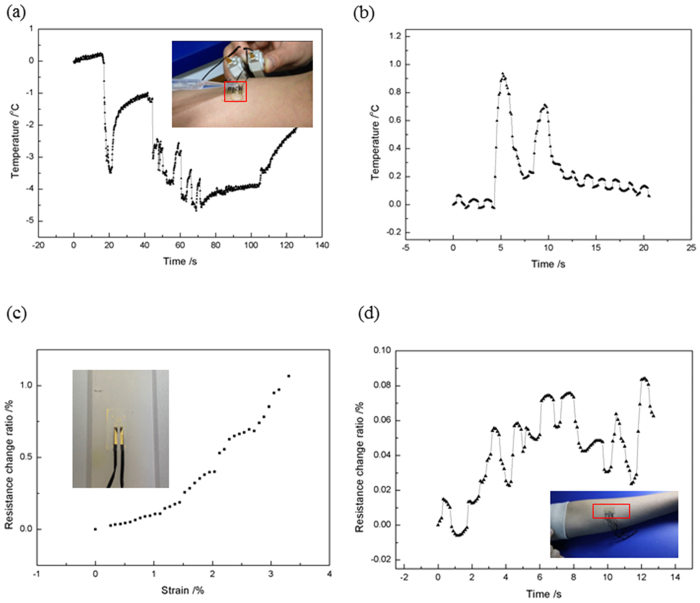
Demonstration of the sensing of water dropping and blowing by mouth, and the noise analysis experiment. (**a**) Temperature change in water dropping (with video in the supporting materials). (**b**) Temperature change in mouth blowing. (**c**) Resistance change under stretching deformation measured with universal tensile testing machine. (**d**) Resistance change while the arm twisting and rotating (with video in the supporting materials).

Besides the body temperature measuring function, this thin and conformal device can be used to sense subtle temperature change on the skin surface caused by the change of its environment, which can be useful in the artificial skin system building for prosthesis or robots. With this sensor, robots will not only able to grab and move things, they are capable of feeling in the change of the environment, such as water dropping and wind blowing in a way of sensing temperature changing. To demonstrate this, the BCTS is located on the inner side of the forearm, with a dropper to create the scenario of raining by dropping the water slowly, as shown in the video in the supplemental material (Supplementary Video 1). The DC resistance meter is connected to measure and record the signal. If the resistance blows up, the device is break down. During the water dropping test the device works normally indicating and confirming that the water proof property of it. As the water dropping on, the temperature on the skin surface locally drops down at the same time, and when the water drop slips off, the temperature reverts by regaining the heat from undisturbed area and inside of the skin. This experience of temperature change process of the local skin surface is delicately sensed by the BCTS. As shown in Fig. 4(a) the temperature variation is in accord with the action of the water dropping on the skin surface, in which 0 °C is referring to the current human body temperature. From the beginning the local body temperature is recovering from the last time of dropping as the signal is going up with a slight slope, then a full water drop reaches the skin surface, the local temperature decreases by ~3.5 °C. When the full water drop flows off, the temperature recovers. Before the temperature returns to normal, several little water drops are followed making the temperature go up and down in smaller amplitude. When the dropping is stopped, the local temperature measured by the BCTS is returning to the normal body temperature. Figure 4(b) demonstrates the temperature change caused by 2 times of blowing to the surface of the skin by mouth. It can be seen that temperature goes up when under blowing with mouth, and goes down when the blow stops. And compared with temperature change caused by water drop, blowing induces smaller temperature change, which can be explained by the temperature difference of the water drop and the moth blowing to the skin surface as well as the difference of heat transmission modes of the two. In blowing test, the heat is transferred by convection, while that of the water dropping is conduction, which is more effective and stronger under this circumstance. And these difference happened on the skin surface can be subtly captured by the BCST, indicating its promising application in smart artificial skin.

The ideal temperature sensor for human body temperature monitoring based on metal temperature resistance effect is that it can be robust with respect to noise, besides owning the advantage of flexibility, stretchability, mechanical invisibility and biocompatibility. The source of the noise are mainly the resistance change caused by piezoresistive effect due to deformation in the device when the working plane deflected or distorted and/or the resistance change caused by the imperfect bonding of the extraction wire to the extraction pad. Figure 4(c) shows the resistance change ratio toward strain relationship of the BCTS in the uniaxial tension test, in which a silicone bar is used as a buffer as shown in the Fig. 4(c) inset. As Fig. 4(c) shows, the resistance change ratio can be up to 1% when the strain is 3.5%, which is far larger than the fracture strain of gold indicating that the stretchable design does works. Still there’s plenty space to be promoted and improved, such as the optimizing of the bonding between the extraction wire and extraction pad. To be more practical, *in vitro* test of the device located on the bending and twisting forearm is performed, as shown in Fig. 4(d) inset. Resistance of the BCTS is measured *in situ* when the forearm is moving and deforming in a random way, with details of the whole process shown in the supplementary video (Supplementary Video 2). During this *in vivo* test, despite of arm’s rigid body movement and tensile/compressive/bending deformation, the device works properly, and the signal is shown in Fig. 4(d). The largest resistance change ratio is ~0.08% corresponding to a noise in temperature with the value of ~0.29 °C. Since both the sensing part and S-shaped wires have similar geometry, the stress/strain state in which is demonstrated by FEM with a representative element under uniaxial stretching. The deformation process on maximum principal strain contour is shown in supplementary video 3. Results show that the element is buckling out of plane to accommodate the stretching load from the substrate, which is in accord with the stretchable design. To the application concern in-bed patients or static resting conditions, this noise can be much smaller and acceptable as expected. For moving and sporting conditions, still there is work to do to lower the noise.

## Conclusion

A biocompatible temperature based on semipermeable substrate aiming at 24/7 health monitoring is designed, fabricated and tested. The porosity structure, small thickness and low modulus, together with excellent elongation of the substrate enable the device to be mechanically invisible and breathable to the wearer when applied on the human skin. The transfer printing in solution is developed to fulfill the fabrication of such thin and stretchable device. Continuous body temperature measuring and 24 hours device wearing test demonstrate the feasibility and reliability of the BCTS to measuring human body temperature. Water dropping and blowing test indicate the device’s ability in reflecting subtle temperature change caused by the surrounding environment, suggesting the possible application of the device to the smart system building for artificial skin. Finally, the design and fabrication method provides the important hints for other wearable and biocompatible devices for long-term health monitoring.

## Methods

### Fabrication of the biocompatible temperature sensor

The fabrication process of BCTS starts with preparation of sacrificial layer of polyimide (PI, ZKPI-3051), in which it is spinning coated on the silicon wafer with the thickness of ~1.5 *μm*, and cured at the temperature of 80 °C for 10 min, 120 °C for 10 min and 140 °C for 30 min. On the PI covered silicon wafer Cr (10 nm)/Au (100 nm) are deposited sequentially by sputtering, in which Cr acts as transition layer and Au the material in the function layer. Then etch Au/Cr into designed patterns by photolithography with the photoresist (PR, AZ5214E) as mask. The semipermeable film (Opsite, Smith & Nephew) is conformed to the patterned wafer as soft targeting substrate before transfer printing in solution. After transfer printing, the patterned ultra-thin metal film is integrated with the soft targeting substrate. Then CNT film ribbons are used as the extraction wire connected with the extraction pad. Finally, encapsulating the sensor with another layer of SF finishes the fabrication process.

### Transfer printing in solution

With polyimide (PI, ZKPI-3051) acting as sacrificial layer, KOH solution (diluted AZ400 K, AZ Electronic Materials, USA) is used to create the liquid environment for transfer printing. The soft targeting substrate is attached to the wafer in a flat and smooth way. An extra layer of relatively stiffer polymer film is used to hold the thin and soft substrate, keeping it from crimping in the etching solution/ DI water after releasing from the wafer or while washing the etching solution off it. When the sacrificial layer is removed, the ultra-thin metal film is released from the silicon wafer and printed to the targeting soft substrate. After the sacrificial layer is full removed, DI water is used to clean the sensor with soft substrate, which finishes the process of transfer printing in solution.

### Calibration of the biocompatible temperature sensor

The sensor is fixed to a thin plate bathed in boiled DI water to rule out the noise of piezoresistive effect. Boiled DI water heated by hot plate is used as temperature changing environment to eliminate the influence of bubbles generated in first heating. The DC resistance meter (TH2515, China) is used to measure and record the resistance of the device. Resistance temperature detector (RTD, CENTER 376, Taiwan) based on platinum (Pt) is employed as temperature reference. The calibration temperature changes from 22 to 45 °C. The slope of resistance-temperature curve represents the TRC of the biocompatible sensor.

### Analysis of the stress in extraction pad at the conjunction of extraction wire

The combination of extraction pad (represented by subscript *pad*) and extraction wire (represented by subscript *wire*) can be regarded as series connected, which share the same force (denoted by *F*) when under a certain load of displacement (denoted by *u*). Considering the combination under stretching, the stress (denoted by σ ) in the extraction pad can be written as 
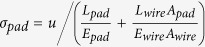
, where *L* is the length under consideration, *E* is Young’s modulus, *A* is cross sectional area.

## Additional Information

**How to cite this article**: Chen, Y. *et al.* Breathable and Stretchable Temperature Sensors Inspired by Skin. *Sci. Rep.* **5**, 11505; doi: 10.1038/srep11505 (2015).

## Supplementary Material

Supplementary Video S1

Supplementary Video S2

Supplementary Video S3

Supplementary Information
