# Supplementary material for: Breathable and Stretchable Temperature Sensors Inspired by Skin
Source: Sci Rep. 2015 Jun 22;5:11505. doi: 10.1038/srep11505 (PMC4476093; doi:10.1038/srep11505)
Supplement: Supplementary Information [file srep11505-s4.pdf]

Breathable and Stretchable Temperature Sensors Inspired by Skin

Ying Chen[1,2], Bingwei Lu[1,2], Yihao Chen[1,2], Xue Feng[1,2]*

[1] Department of Engineering Mechanics, Tsinghua University, Beijing 100084, China;
[2] Center for Mechanics and Materials, Tsinghua University, Beijing 100084, China

* Corresponding author, Xue Feng, fengxue@tsinghua.edu.cn

Supplementary Video 1. The demonstration of water proof property of the BCTS and its ability to sense the environment changes, such as water dropping, by detecting the slight temperature change.

Supplementary Video 2. The demonstration of in situ temperature measuring by putting the BCTS on the forearm. The device works properly despite of arm's rigid body movement and tensile/compressive/bending deformation.

Supplementary Video 3. The FEM results of the representative element in uniaxial stretching. The video shows the deformation (deformation scale factor=10) of the S-shaped element on contour of maximum principal strain distribution.
